# Discovery of New Hits as Antitrypanosomal Agents by In Silico and In Vitro Assays Using Neolignan-Inspired Natural Products from *Nectandra leucantha*

**DOI:** 10.3390/molecules26144116

**Published:** 2021-07-06

**Authors:** Sheila C. Araujo, Fernanda S. Sousa, Thais A. Costa-Silva, Andre G. Tempone, João Henrique G. Lago, Kathia M. Honorio

**Affiliations:** 1 Centro de Ciências Naturais e Humanas, Universidade Federal do ABC, Avenida dos Estados, 5001 Bangu, Santo André 09210-580, SP, Brazil; sheila.araujo@alumni.usp.br (S.C.A.); tha_isbio@yahoo.com.br (T.A.C.-S.); 2 Instituto de Ciências Ambientais, Químicas e Farmacêuticas, Universidade Federal de São Paulo, Rua Prof. Arthur Riedel, 275, Diadema 09972-271, SP, Brazil; fesamara@gmail.com; 3 Departamento de Fisiologia e Biofísica, Universidade Federal de Minas Gerais, Avenida Presidente Antônio Carlos, 6627, Belo Horizonte 31270-901, MG, Brazil; 4 Centre for Parasitology and Mycology, Instituto Adolfo Lutz, Avenida Doutor Arnaldo, 351, São Paulo 01246-000, SP, Brazil; andre.tempone@ial.sp.gov.br; 5 Escola de Artes, Ciências e Humanidades, Universidade de São Paulo, Rua Arlindo Bettio, 1000 Ermelino Matarazzo, São Paulo 03828-000, SP, Brazil

**Keywords:** Chagas disease, *Trypanosoma cruzi*, neolignans, drug design, in silico, in vitro assays

## Abstract

In the present study, the phytochemical study of the *n*-hexane extract from flowers of *Nectandra leucantha* (Lauraceae) afforded six known neolignans (**1**–**6**) as well as one new metabolite (**7**), which were characterized by analysis of NMR, IR, UV, and ESI-HRMS data. The new compound **7** exhibited potent activity against the clinically relevant intracellular forms of *T. cruzi* (amastigotes), with an IC_50_ value of 4.3 μM and no observed mammalian cytotoxicity in fibroblasts (CC_50_ > 200 μM). Based on the results obtained and our previous antitrypanosomal data of 50 natural and semi-synthetic related neolignans, 2D and 3D molecular modeling techniques were employed to help the design of new neolignan-based compounds with higher activity. The results obtained from the models were important to understand the main structural features related to the biological response of the neolignans and to aid in the design of new neolignan-based compounds with better biological activity. Therefore, the results acquired from phytochemical, biological, and in silico studies showed that the integration of experimental and computational techniques consists of a powerful tool for the discovery of new prototypes for development of new drugs to treat CD.

## 1. Introduction

**Figure 1:**
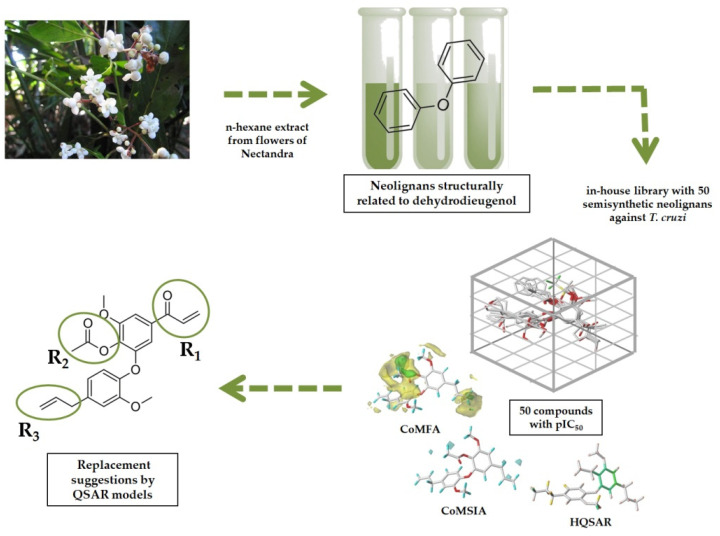
Workflow used to study new neolignan-based compounds with potential trypanocidal activity from *N*. *leucantha*.

Protozoan parasitic diseases affect a large population in tropical and subtropical regions worldwide. In particular, underdeveloped and developing countries in Asia and Latin America concentrate the highest rates of morbidity and mortality [1]. Chagas disease (CD) is caused by the parasite *Trypanosoma cruzi* and is a challenging parasitic disease, relying on two toxic drugs for treatment, nifurtimox and benznidazole [2,3]. Considering the urgent need for new treatments, natural products can be considered important sources to design potent and safer drug candidates [4,5,6,7]. Different studies [8,9,10] have shown that neolignans isolated from leaves and twigs of *Nectandra leucantha* (Lauraceae), as well as several related semi-synthetic derivatives, exhibited in vitro activity against *T. cruzi* [1,8,11]. In this work, molecular modeling techniques were employed to aid in the design of new neolignan-based compounds with improved biological activity against *T. cruzi*. It is noteworthy to mention that, from molecular modeling studies and statistical models, valuable results have been obtained taking into account libraries of bioactive substances related to several biological targets [12,13,14,15]. In particular, computational techniques directed to studies related to CD can be considered as powerful tools to help in the discovery and the design of selective, potent, and safer drug candidates [16,17,18,19]. In this study, seven bioactive neolignans (**1**–**7**) were isolated from the flower extract of *N. leucantha* and were, altogether with other related bioactive compounds (**8**–**50**), subjected to robust and predictive in silico models, using two-(2D) and three-(3D) dimensional techniques [20,21,22,23,24], as indicated in Figure 1. It is also worth noting that the use of various techniques to construct predictive models is an important strategy since these approaches employ different and complementary molecular information (for example, bidimensional, steric/electrostatic/hydrophobic features), which helps in the design of new drug candidates.

## 2. Results and Discussion

### 2.1. Chemical Characterization of Compounds ***1***–***7*** Isolated from Flowers of N. leucantha

The phytochemical analysis of the flower extract from *N. leucantha* afforded seven related neolignans, six of which are known: Dehydrodieugenol B (**1**), 1-(8-propenyl)-3-[3′-methoxy-1′-(8-propenyl)-phenoxy]-4,5-dimethoxybenzene (**2**), 1-[(7*S*)-hydroxy-8-propenyl]-3-[3′-methoxy-1′-(8′-propenyl)-phenoxy]-4-hydroxy-5-methoxybenzene (**3**), 1-[(7*S*)-hydroxy-8-propenyl]-3-[3′-methoxy-1′-(8′-propenyl)-phenoxy]-4,5-dimethoxybenzene (**4**), 4-hydroxy-5-methoxy-3-[3′-methoxy-1′-(8′-propenyl)phenoxy]-1-(7-oxo-8-propenyl)benzene (**5**), and 4,5-dimethoxy-3-[3′-methoxy-1′-(8′-propenyl)phenoxy]-1-(7-oxo-8-propenyl)benzene (**6**), as well as one new natural product (**7**). Structures of compounds **1**–**6** were identified by comparison of reported NMR and ESI-HRMS data to the neolignans previously isolated from leaves and branches of *N. leucantha* [4,8,25].

Compound **7** was isolated as a white amorphous solid. Its IR spectrum showed bands characteristic of carbonyl groups at 1732 cm (ester) and at 1680 cm^−1^ (conjugated ketone) and double bonds of an aromatic ring at 1681 cm^−1^ and C-O at 1153 cm^−1^. ^1^H NMR spectrum of compound **7** exhibited similarities to those recorded from related compounds **5** and **6** including the signals at δ 7.45 (d, *J* = 1.8 Hz) and δ 7.32 (d, *J* = 1.8 Hz), assigned to aromatic hydrogens H-2 and H-6 and at δ 6.82 (d, *J* = 1.8 Hz), 6.95 (d, *J* = 8.1 Hz), and 6.76 (dd, *J* = 8.1 and 1.8 Hz), attributed to H-2′, H-5’ and H-6´, respectively [25]. As this spectrum showed an ABX system at δ 7.01 (dd, *J* = 17.1 and 10.6 Hz, H-8), 6.37 (dd, *J* = 17.1 and 1.8 Hz, H-9a), and 5.82 (dd, *J* = 10.6 and 1.8 Hz, H-9b), the occurrence of an acryloyl group at C-1 was proposed. One intense singlet at δ 2.01 (3H) was also observed, suggesting the presence of an acetyl group. The ^13^C NMR spectrum showed, besides the aromatic signals at range δ 152–107, the presence of peaks at δ 192.3, 131.7, and 129.5, which were attributed to C-7, C-8, and C-9, respectively, confirming the presence of an acryloyl group at C-1. Additional signals at δ 168.9 and 20.1 were assigned to an acetoxyl group at C-4 while those at δ 40.0 (C-7′), 137.3 (C-8′), and 116.2 (C-9′) indicated the presence of an allyl side chain at C-1′ [1,25]. Furthermore, the ESI-HRMS spectrum displayed the [M + H]^+^ ion peak at *m*/*z* 383.1490, in agreement with the molecular formula C_22_H_22_O_6_. Finally, the observed spectral data to the natural product **7** (Supplementary Material) were identical to those recorded for the acetylated derivative of compound **5**. Therefore, the structure of compound **7** was defined as 4-acetoxy-5-methoxy-3-[3′-methoxy-1′-(8′-propenyl)phenoxy]-1-(7-oxo-8-propenyl) benzene.

### 2.2. Anti-T. cruzi Activity of the Natural Products ***1***–***7***

Compounds **1**–**7** were evaluated against *T. cruzi* (in vitro) using intracellular amastigote forms of the parasite. As shown in Table 1, compounds **1**–**3** were inactive, whereas compound **4** exhibited a moderate IC_50_ value (14.3 µM) when compared to the standard drug benznidazole (5.5 µM), but with reduced mammalian cytotoxicity (CC_50_ > 200 µM). Compounds **6** and **7** exhibited IC_50_ values of 26.3 and 4.2 µM, respectively, indicating that **7** displayed a superior potential in comparison with natural products **1**–**6** (Table 1). Based on these results, it was observed that the presence of α,β-unsaturated carbonyl system in the side chain (position C-1) plays an important role in the bioactivity, along with the presence of acetoxyl group at the aromatic ring (position C-4). It is important to note that compound **5**, which exhibits one free phenol group, was inactive (IC_50_ > 30 µM) and compound **6**, that contains one methoxyl group at the same position, exhibited a moderate potential. Similar to the compounds with allyl side chain and hydroxyl (compound **1**) and methoxyl (compound **2**), both with IC_50_ > 30 µM, the activity against the intracellular amastigotes was intensified with an acetoxyl moiety at the aromatic ring (semi-synthetic compound **12** displayed expressive activity with an IC_50_ value of 8.0 µM). Regarding studies with fibroblasts, all tested natural compounds exhibited no mammalian cytotoxicity, with CC_50_ values higher than 200 µM. Finally, the selectivity index (SI) for the most active compound **7** (SI > 47.6) was also superior to that of the standard drug benznidazole (SI = 34.5), demonstrating a promising potential for this new natural product. It is interesting to note that the values of pIC_50_ predicted by the in silico models are very similar to the experimental ones, indicating the predictive power of the models obtained in this study. Therefore, the in silico and the experimental results suggest that compound **7** can be considered a good hit compound for the design of new candidates for Chagas disease.

### 2.3. Dataset Used In Silico Analyses

**Figure 2:**
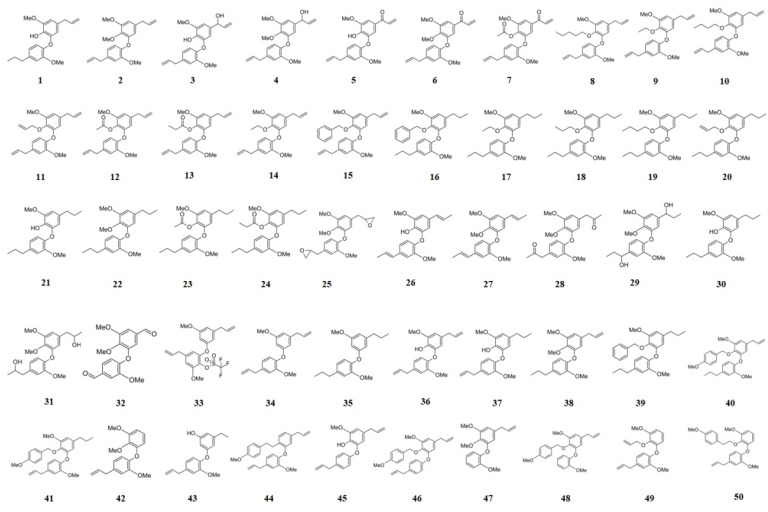
Chemical structures of natural (**1**–**7**) and semisynthetic (**8**–**50**) neolignans.

**Figure 3:**
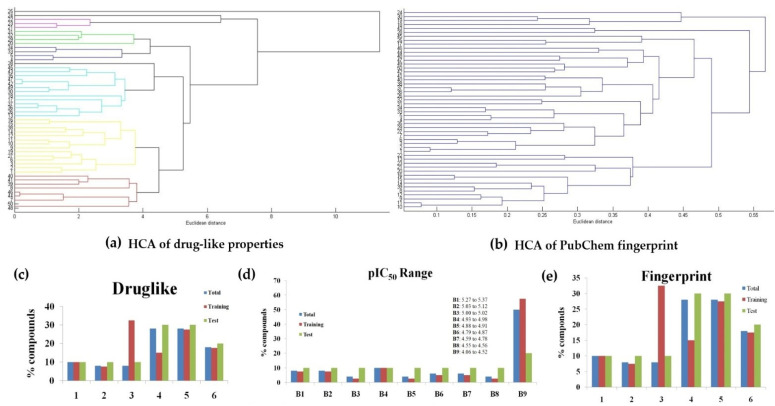
HCA results (dendrogram) for the compound set obtained from (**a**) drug-like properties and (**b**) molecular fingerprint. Distribution of compounds in training, test, and total sets according to (**c**) drug-like properties, (**d**) pIC_50_ range, and (**e**) molecular fingerprint.

Hereafter, natural products **1**–**7** and related semisynthetic neolignans **8**–**50** were used to construct 2D and 3D predictive models. For this, the compound set used in the present study (Figure 2) was divided in training (40 compounds) and test (10 compounds) sets. For the selection of the training and test sets, HCA (Hierarchical Cluster Analysis) was used to organize the molecules in groups according to their similarities from drug-like and fingerprint properties in the form of a dendrogram by using Euclidean distance. The test set was selected from the remaining representative structures from each one of the observed clusters considering information on drug-like properties, molecular fingerprint, and pIC_50_ ranges (Figure 3).

### 2.4. Molecular Alignment

**Figure 4:**
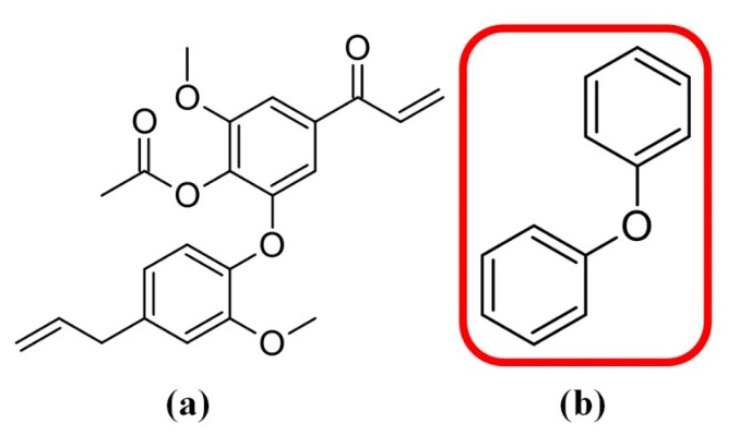
Maximum common structure (MCS) considered for the alignment of the molecules: (**a**) Compound **7** (the most active of the series) was used as a template; (**b**) general structure of the neolignans with the core used in the molecular alignment.

**Figure 5:**
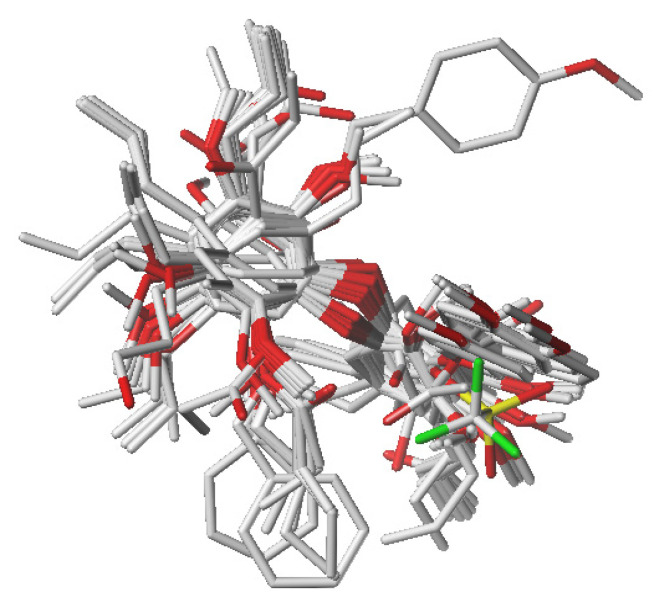
Alignment of the compound set using the maximum common substructure (MCS) obtained from Distill.

To construct the 3D models, a molecular alignment of the compound set was obtained from a lattice grid [27]. In this study, the molecular alignment of the neolignans **1**–**50**, tested against *T. cruzi*, was based on the most active compound **7** that was used as a template (see Figure 4a). To align the molecules, it was necessary to define a core (Figure 4b), and the RMS (Root Mean Square) deviation was examined to generate a single mapping. The molecular alignment obtained in this study is displayed in Figure 5.

### 2.5. HQSAR Modeling

Table 2 presents the main statistical results obtained from the HQSAR (Hologram QSAR) technique. Among all the generated HQSAR models (Table 2), the 2D best models obtained for the studied neolignans showed *q*^2^_LOO_ between 0.68 and 0.75. The model with the best statistical parameters had atoms, bonds, hydrogens, chirality, and hydrogen bond donors and acceptors (A/B/H/Ch/DA, with *q*^2^_LOO_ = 0.75) as the best fragment distinction. It is important to note that the addition of the options “Chirality” and “H bond donor-acceptor” significantly improved the statistical quality of the 2D model. Furthermore, the best previous model (highlighted in Table 2) was submitted to variation of the fragment size to check improvements in the statistical quality of the model (see Table 3).

From Table 3, it is possible to observe a statistical improvement in the models after the parameter optimization (fragment size). So, this better model (according to the *q^2^*_LOO_ values) was selected and used to better understand the molecular clues related to the biological activity of the studied neolignans.

### 2.6. CoMFA and CoMSIA Models

3D statistical models were generated using CoMFA (Comparative Molecular Field Analysis) and CoMSIA (Comparative molecular similarity indices analysis), implemented in Sybyl 8.1 [27]. The main statistical parameters for all CoMFA (Table 4) and CoMSIA (Table 5) models were obtained by varying the standard settings initially. Afterwards, the option “regions focus” was used to refine the statistical parameters. The statistical quality of the models was analyzed according to the correlation coefficients (*q*^2^ obtained from the cross-validation, and *r*^2^), the number of principal components (PC), and other parameters such as standard error of estimation (SEE). The predictive CoMFA and CoMSIA models are the ones with minimal PC from the cross-validated PLS regression, which were also used to generate the contour maps.

The maximum number of PC used in both CoMFA (Table 4) and CoMSIA (Table 5) models respected the size of the dataset (compounds **1**–**50**) and were sufficient to explain the variability of the system under study. The best CoMFA and CoMSIA models were, respectively, selected from the internal (*q*^2^_LOO_ > 0.80) and external (Q^2^_F3_ and Q^2^_F3_ > 0.75) robustness. From these models, the contour maps for some compounds of the dataset (the most and least active ones) were generated. Other metrics for the external validation were also used to assess the predictive power of the 3D models [28]. The sensitivity index ($dq^{2}/dr^{2yy\prime }$) was generated by 25 runs of progressive scrambling for the CoMFA and CoMSIA analyses (the expected values of this index should be between 0.8 and 1.2—see Table 6). In addition, the applicability domain was determined (see Supplementary Material, Figure S1) indicating that all compounds (training and test) are at the left-bottom dashed-lined quadrant of Leverage and Studentized residual, suggesting there is no outlier sample. Tests to check chance correlations from progressive scrambling (CoMFA and CoMSIA) and Y-scrambling (Table 6) were also performed.

### 2.7. External Validation

**Figure 6:**
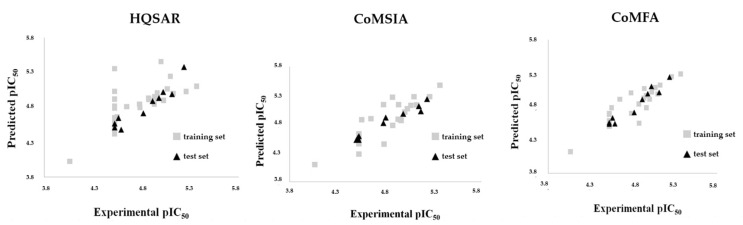
Experimental and predicted pIC_50_ for the compound sets (training and test) obtained from the HQSAR, CoMFA, and CoMSIA models. Grey squares: Training set; black triangles: Test set.

Although the HQSAR, CoMFA, and CoMSIA models complement each other because they are trained with a lot of chemical information from the training set, it is necessary to perform external validation. So, the HQSAR, CoMFA, and CoMSIA models were externally validated to prove their statistical quality (predictive power) based on OECD (Organization for Economic Cooperation and Development) guidelines [28]. From the obtained results, the 2D and 3D models can be considered predictive due to *r*^2^*_pred_* values >0.8 (see Figure 6 and Table 7). The high Q^2^_F2_ and Q^2^_F3_ values suggest that the CoMFA [29] and CoMSIA models have predictive ability. Residuals between predicted and experimental activity were always smaller than 1 log unit (Table S1, Supplementary Material).

To test the statistical performance of the models according to variations in the composition of the training set, a leave-N-out (LNO) validation (Figure S2, Supplementary Material) with different numbers of cross-validation groups (5 to 15) was also performed. The average values of *q*^2^ were bigger than 0.7 indicating a great internal consistency. In sum, the best 2D and 3D models (*q*^2^_LOO_ > 0.6) were submitted to external validation (test set). Table 7 displays the main results obtained from the external validation and Table 1 displays a summary of the main biological parameters for the natural (**1**–**7**) and semisynthetic (**8**–**50**) neolignans, as well as the predicted pIC_50_ values and residuals obtained from 2D and 3D models.

### 2.8. Physicochemical Interpretation of the Models

**Figure 7:**
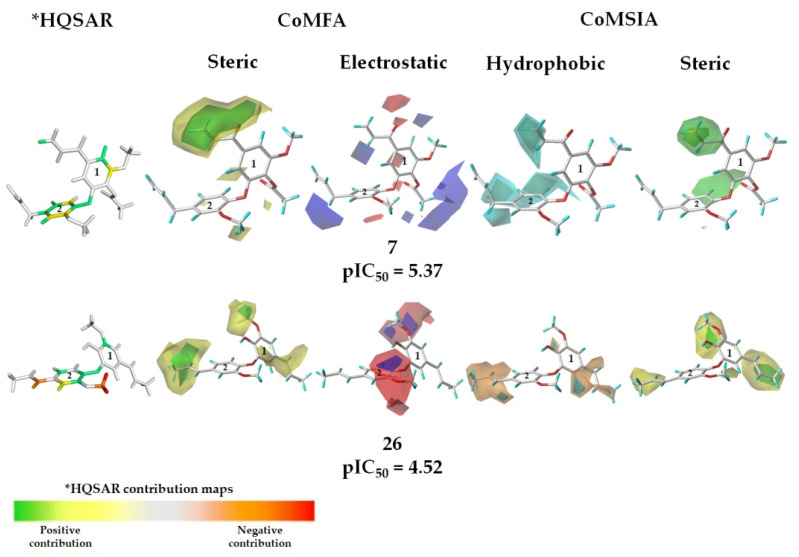
2D and 3D maps for the more active (**7**) and one of the less active compounds (**26**). Green and yellow regions in the HQSAR maps represent substitutions by voluminous groups that can improve the biological activity; orange and red indicate voluminous substituents that can contribute negatively. In addition, green contours in the CoMFA maps suggest that bulky groups can contribute to the biological activity; yellow contours = voluminous groups could decrease the biological activity. In blue regions, positively charged groups can increase the biological property; red regions = negatively charged groups can improve the activity. For the hydrophobic contour maps (CoMSIA): Cyan contours = hydrophobic substituents can enhance the activity; orange contours = hydrophobic groups can decrease the activity. From the CoMSIA maps, green contours suggest that bulky groups can contribute to the biological activity; yellow contours = voluminous groups could decrease the biological activity (favorable contours are set to 80% and the unfavorable regions are set to 20%).

After the construction and the validation of the 2D and 3D models, information on regions that can suffer molecular modifications was obtained from the contribution (2D) and contour maps (from 3D techniques). Figure 7 displays the 2D and 3D maps for the more active compound **7** and one of the less active compounds **26**.

As shown in Figure 7, it is possible to observe that some carbon atoms at the aromatic rings (A and B) are colored green and yellow (from the HQSAR maps) suggesting that these fragments could be important in explaining the biological activity of the analyzed compounds. In particular, the 2D contribution map (HQSAR) for the least active compound (**26**) showed red and orange colors at the allyl group, which could suffer molecular modifications to improve the activity. The steric and electrostatic contour maps, obtained from CoMFA, for compounds **7** and **26** are also shown in Figure 7. In the CoMFA steric maps, there is a region in green and a considerable yellow contour covering the acetoxyl group at ring A in compound **7**. This indicates that replacements by moderately bulky groups close to the acetoxyl group could improve its anti-*T. cruzi* activity. In addition, from the map of compound **7,** a yellow contour close to the allyl group at the aromatic rings A and B was observed, indicating that less voluminous groups in these regions could positively influence the biological response. Therefore, the acetoxyl moiety at the studied neolignans should be changed by substituents with moderate volume (e.g., a tetrahydrofuran moiety).

In the CoMFA electrostatic maps (Figure 7), the blue contour at the allyl group (at the aromatic ring 2) of compound **7** indicates the importance of electropositive groups in this region for the biological activity. Besides, the red contour at the methoxyl group (compounds **7** and **26**) suggests that electronegative substituents can be beneficial to the biological activity. From the CoMSIA technique, hydrophobic and steric contribution contour maps for compounds **7** and **26** were also analyzed (Figure 7). The cyan contours at the allyl and propyl groups at the aromatic rings indicate that hydrophobic groups in these regions could increase the biological activity of tested compounds. From this, one could suggest that compounds **7** and **26** with hydrophobic substituents at the regions cited above and combined with steric contribution groups could improve their biological activity. Besides, the cyan contour indicates the importance of hydrophobic groups, such as an alkyl moiety, to improve the trypanocidal activity of these related neolignans.

**Figure 8:**
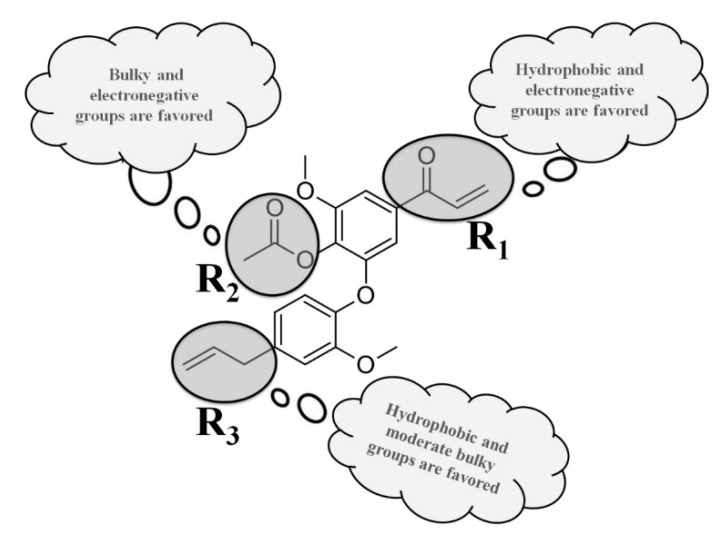
Structure-activity relationships revealed from the HQSAR, CoMFA, and CoMSIA studies applied to compound **7** of the dataset.

The CoMSIA maps related to the steric contributions indicate regions that should have bulky groups to improve the interactions of the ligands with the biological target and, consequently, increasing the biological activity. From Figure 7, it was possible to observe an intense green contour at the acetoxyl moiety in compound **7** and three yellow contours in compound **26**. In addition, there is a green contour in compound **26**, indicating that moderately bulky groups, for example, tetrahydrofuran or pyridine (also containing carbonyl group) could improve the anti-*T. cruzi* amastigote activity of new compounds, which is in agreement with the steric map. The main molecular relationships obtained from all 2D and 3D models are summarized in Figure 8.

## 3. Material and Methods

### 3.1. General Experimental Procedures

Silica gel (60–210 μm—Merck, Darmstadt, Germany) or Sephadex LH-20 (Aldrich, St Louis, MI, USA) were used for column chromatography while silica gel F254 (Merck, Darmstadt, Germany) was used for analytical TLC. UV spectra were recorded on an UV/visible Shimadzu 1650-PC spectrophotometer (Kyoto, Japan). IR spectra were recorded on a Perkin-Elmer 1750 spectrophotometer (Waltham, MA, USA). ^1^H and ^13^C NMR spectra were recorded on a Bruker Ultrashield (Billerica, MA, USA) 300 model Avance III, operating at 300 and 75 MHz, respectively, using CDCl_3_ as solvents and TMS as internal standard. ESI-HRMS spectra were measured on a Bruker Daltonics q-TOF maxis 3G spectrometer (Billerica, MA, USA) operating on electrospray ionization in positive mode.

### 3.2. Isolation of Neolignans ***1***–***7*** from n-Hexane Extract from Flowers of N. leucantha

Fresh flowers of *Nectandra leucantha* (Lauraceae) were collected in Cubatão city (São Paulo state, Brazil), in March 2019, from a previously investigated specimen [11]. After air-drying, *N. leucantha* flowers (55 g) were powdered and exhaustively extracted with *n*-hexane (10 × 250 mL). Combined organic extracts were concentrated under vacuum to afford 1.7 g of a syrup-like material. Part of this material (1.5 g) was subjected to column chromatography over SiO_2_ using increasing amounts of EtOAc in *n*-hexane to afford seven fractions (A–G). Fractions B (612 mg) and D (403 mg) were composed, respectively, by pure **2** and **1**. Part of fraction C (104 mg) was purified by SiO_2_ column eluted with *n*-hexane:EtOAc at 8:2, 7:3, and 1:1 to afford compounds **6** (7.3 mg) and **7** (1.1 mg). Part of fraction F (88 mg) was chromatographed over Sephadex LH-20 and eluted with MeOH to give five fractions (F1–F5). Fraction F2 was composed of pure compound **5** (4.1 mg). Fraction G (24 mg) was purified by prep. TLC (*n*-hexane:EtOAc 8:2) to afford **3** (8.3 mg) and **4** (6.3 mg)

*4-Acetoxy-5-methoxy-3-[3′-methoxy-1′-(8′-propenyl)phenoxy]-1-(7-oxo-8-propenyl)benzene* (**7**). White amorphous solid. UV (MeOH) λ_max_ (log ε) 212 (3.5), 286 (3.1); IR (film) ν_max_ 3350, 2842, 1732, 1681, 1512, 1452, 1153, 965 cm^−1^. ^1^H NMR (CDCl_3_, 300 MHz) δ 7.45 (d, *J* = 1.8 Hz, H-2), 7.32 (d, *J* = 1.8 Hz, H-6), 7.01 (dd, *J* = 17.1 and 10.6 Hz, H-8), 6.95 (d, *J* = 8.1 Hz, H-5′), 6.82 (d, *J* = 1.8 Hz, H-2′), 6.76 (dd, *J* = 8.1 and 1.8 Hz, H-6′), 6.37 (dd, *J* = 17.1 and 1.8 Hz, H-9a), 6.00 (m, H-8′), 5.82 (dd, *J* = 10.6 and 1.8, H-9b), 5.10 (m, H-9′), 3.99 (s, 5-OCH_3_), 3.86 (s, 3′-OCH_3_), 3.38 (d, *J* = 6.6 Hz, H-7′), 2.01 (s, CH_3_ acetyl). ^13^C NMR (CDCl_3_, 75 MHz) δ 192.3 (C-7), 168.9 (C=O acetyl), 152.5 (C-5), 150.5 (C-3′), 150.1 (C-3), 143.2 (C-4′), 137.3 (C-8′), 136.6 (C-4), 135.1 (C-1), 131.7 (C-8), 129.5 (C-9), 121.1 (C-6′), 120.0 (C-5′), 116.2 (C-9′), 113.1 (C-2′), 112.0 (C-2), 106.8 (C-6), 56.7 (5-OCH_3_), 56.0 (3′-OCH_3_), 40.0 (C-7′), 20.1 (CH_3_ acetyl); ESI-HRMS *m*/*z* 383.1490 [M + H]^+^ (calculated for C_22_H_23_O_6_, 383.1495).

### 3.3. Experimental Bioassays

BALB/c mice were obtained by the animal breeding facility at the *Instituto Adolfo Lutz* (São Paulo State, Brazil). The animals were maintained in sterilized boxes with absorbent material under a controlled environment and received water and food ad libitum. BALB/c mice were used to obtain peritoneal macrophages. Animal procedures were performed with the approval of the Research Ethics Commission (project CEUA-IAL 05/2018), in agreement with the Guide for the Care and Use of Laboratory Animals from the National Academy of Sciences.

### 3.4. Parasites and Mammalian Cell Maintenance

*T. cruzi* trypomastigotes (Y strain) were maintained in Rhesus monkey kidney cells (LLC-MK2-ATCC CCL 7), using RPMI-1640 medium supplemented with 2% FBS (Fetal Bovine Serum) at 37 °C in a 5% CO_2_-humidified incubator. The murine conjunctive cells (NCTC clone 929, ATCC) and LLC-MK2 were maintained in RPMI-1640 supplemented with 10% FBS at 37 °C in a 5% CO_2_-humidified incubator. Macrophages were obtained from the peritoneal cavity of BALB/c mice by washing them with RPMI-1640 medium supplemented with 10% FBS and were maintained at 37 °C in a 5% CO_2_-humidified incubator.

### 3.5. Determination of Activity against Amastigote Forms of T. cruzi

The 50% inhibitory concentrations (IC_50_) of compounds **1**–**7** were determined against amastigotes forms of *T. cruzi*. To perform the experiments, peritoneal macrophages from BALB/c mice were infected with trypomastigotes forms of *T. cruzi*. The macrophages obtained from the peritoneal cavity of BALB/c mice were seeded on a 16-well chamber slide (NUNC plate, Merck; 1 × 10^5^/well) and incubated for 24 h at 37 °C in a 5% CO_2_-humidified incubator. The trypomastigotes LLC-MK2 were counted and used to infect the macrophages (parasite:macrophage ratio = 10:1). After 2 h incubation at 37 °C in a 5% CO_2_-humidified incubator, free parasites were removed by 1× washing with the medium. Next, tested compounds were incubated with infected macrophages for 48 h at 37 °C, 5% CO_2_ in a range concentration of 30 to 0.94 µM. Benznidazole was used as the standard drug. At the end of the assay, slides were fixed with MeOH, stained with Giemsa, and counted using light microscopy. The IC_50_ values were calculated as previously reported [30].

### 3.6. Determination of Cytotoxicity against Mammalian Cells

The cytotoxicity of compounds **1**–**7** was determined against NCTC cells-clone L929. The cells (6 × 10^4^ cells/well) were seeded and incubated with the tested compounds (200–1.56 µM) for 48 h at 37 °C in a 5% CO_2_ incubator. The 50% cytotoxic concentration (CC_50_) was determined by MTT assay [31]. The optical density was determined using FilterMax F5 (Molecular Devices) at 570 nm. We calculated the selectivity index (SI) values using the following equation: CC_50_ against NCTC cells/IC_50_ against amastigotes.

### 3.7. Compound Set

An in-house library containing fifty neolignans (natural and semi-synthetic), tested against amastigote forms of *T. cruzi,* was selected for this study (Figure 2) [1,10]. The anti-amastigote data were converted into pIC_50_ (-log IC_50_), taking into account the value of IC_50_ against amastigotes (Figure 2). The 2D structures of compounds **1**–**50** were drawn using MarvinSketch 15.8.31 [32], while the 3D structures were drawn in Avogadro [33]. Afterwards, a conformational analysis of these structures was carried out with the Hartree–Fock method (HF) [34], available in Gaussian09 [35]. The compounds from the dataset were divided in training and test sets taking into account some molecular and biological parameters, such as drug-like properties, molecular structural diversity, and ranges of biological activity using PaDel [36]. For the clustering of the compounds, hierarchical cluster analysis (HCA) implemented at Chemoface [37] was employed. It is important to mention that there are several other computational algorithms used to perform HCA and other statistical analyses, for example, the tool available at https://dtclab.webs.com/software-tools (accessed on 20 April 2021). The HCA method was used to explore the organization of samples in groups according to their drug-like properties and fingerprints depicting classifications based on their physicochemical properties. The result is presented as a dendrogram that shows the organization of the samples and their relationships according to Euclidean distance. Information on the molecular structures was encoded from fingerprints available at PubChem. The drug-like properties used in this analysis were topological polar surface area (TPSA), number of H-bond acceptors (HBA) and donors (HBD), clogP, number of rotatable bonds (nRotB), and molecular weight (MW). Fingerprints and all descriptors [36] were normalized before the HCA analyses (see Supplementary Material—Table S2). In silico models were generated using three independent approaches: HQSAR (hologram quantitative structure–activity relationships), CoMFA (comparative molecular field analysis), and CoMSIA (comparative molecular similarity indices analysis). All of the models were constructed from the training set and using the partial least-squares (PLS) technique, available at Sybyl 8.1 [27].

### 3.8. Rigid Alignment of the Compound Structures

For all 3D analyses (CoMFA and CoMSIA), the rigid alignment of the selected compounds **1**–**50** (Figure 2) was performed by using Sybyl 8.1 and the approach was based on the maximum common substructure (MCS) [27]. This kind of molecular alignment was used because the compounds studied have no known biological targets.

### 3.9. HQSAR Modeling

Hologram QSAR (HQSAR) is a technique that uses bidimensional molecular information and relates this information to biological activity for a set of related compounds. The generation of the HQSAR model may be affected by several parameters: Fragment size (FS), hologram length (HL), and fragment distinction (FD). Hence, different HQSAR runs were considered varying the following parameters: Default hologram lengths (53, 59, 61, 71, 83, 97, 151, 199, 257, 307, 353, and 401 bins), fragment sizes (1–4, 2–5, 3–6, 4–7, 5–8, 6–9, and 7–10 atoms), and different combinations of FD parameters (atoms, bonds, connections, hydrogen atoms, chirality, and hydrogen bond donor/acceptor atoms) [27,38]. Next, the 2D model was constructed by using PLS that relates the hologram descriptors and the biological data.

### 3.10. Generation of CoMFA and CoMSIA Models

CoMFA and CoMSIA techniques employ probe atoms (in general, a positively charged sp^3^ carbon) to obtain molecular interaction fields and a range of different similarity indices, respectively, that are related to the biological data [26,39]. To construct CoMFA and CoMSIA models, we used the molecular alignment based on the technique known as maximum common substructure (MCS) of the training molecules (grid spacing = 2 Å, and energy cut-off = 30 kcal/mol) [26,39]. To construct the CoMFA models, we calculated molecular interaction (steric and electrostatic) fields by using a probe atom from the Lennard–Jones and Coulomb potentials, respectively [26]. Regarding the CoMSIA models, different similarity indices (steric, electrostatic, hydrophobic, H-bond donor, and H-bond acceptor) were obtained. To select the best models, the values of *q*^2^_LOO_ were analyzed and the selected models were also optimized using the option “region focus”. It is important to mention that the grid spacing was varied (1 to 4 Å), as well as the weight factor (from 0.3 to 1.5) [26].

### 3.11. Validation of the Statistical Models

Internal (leave-one-out and leave-N-out cross-validations) and external (using a test set) validations were performed to assess the statistical quality of all models (HQSAR, CoMFA, and CoMSIA). The best statistical models obtained (HQSAR, CoMFA, and CoMSIA) were robust due to the values of *q*^2^_LOO_ (internal validation). Other metrics were also employed to guarantee the predictive power of all obtained models, for example, *r*^2^_m_ [28,40,41]. Moreover, we also considered the principles advocated by OECD to develop reliable and predictive models [28,42]. It is important to note that the models were also submitted to tests to check for chance correlations (progressive scrambling).

## 4. Conclusions

The present study indicates, for the first time, the anti-*T. cruzi* (amastigote forms) potential of new compound **7**, a dehydrodieugenol B derivative isolated from flowers of *N. leucantha*. Based on the results obtained, it was possible to conclude that compound **7**, exhibiting important structural features such as acetoxyl, allyl, and acryloyl groups could be considered a promising scaffold molecule to be used as a prototype for the development of new drugs for Chagas disease therapy. From the construction and the validation of the 2D and 3D (HQSAR, CoMFA, and CoMSIA) models for the neolignan set, we can conclude that the models are statistically robust and have a good predictive power, indicating that they can be employed to predict the biological property of new compounds against amastigote forms of *T. cruzi*. From the present work, it is possible to propose molecular modifications in the neolignan derivatives to obtain new compounds with improved biological activity. As a result, it was possible to observe that the R_1_ position requires moderately hydrophobic and electronegative groups. In addition, the presence of moderately bulky groups at R_2_ is important for biological activity. The analyses carried out from all constructed models contributed to the discovery of R_1_ and R_2_ positions as the best regions to perform in silico replacements for trypanocidal activity improvement of new neolignan-based compounds. Therefore, the experimental and in silico techniques employed in this study were important tools to understand the main molecular features of the neolignans related to the biological activity under study.

## Figures and Tables

**Table 1:** Anti-*T. cruzi* activity from in vitro assays and in silico results of natural (**1**–**7**) and semisynthetic (**8**–**50**) neolignans ^1^.

	IC_50_ (μM) ^2^	CC_50_ (μM) ^3^	SI ^4^	pIC_50_ Experimental	pIC_50_				Residual	
					HQSAR	CoMFA	CoMSIA	HQSAR	CoMFA	CoMSIA
**1**	>30	>200	-	4.52	4.60	4.53	4.47	0.08	0.01	0.05
**2**	>30	>200	-	4.52	4.80	4.53	4.57	0.28	0.00	0.05
**3**	>30	>200	-	4.52	4.57	4.52	4.58	0.05	0.00	0.05
**4**	14.3 ± 1.9	>200	>14.0	4.84	4.72	4.71	4.78	0.13	0.14	0.07
**5**	>30	>200	-	4.52	4.46	4.56	4.51	0.06	0.04	0.01
**6**	26.3 ± 1.3	>200	>7.6	4.58	4.65	4.63	4.51	0.07	0.05	0.07
**7**	4.2 ± 1.1	>200	>47.6	5.38	5.10	5.29	5.48	0.27	0.09	0.10
**8**	>30	>200	-	4.52	4.66	4.78	4.87	0.11	0.26	0.35
**9**	16.4 ± 2.1	>200	>12.2	4.79	4.80	4.69	4.45	0.01	0.09	0.33
**10**	>30	>200	-	4.52	4.52	4.53	4.48	0.00	0.01	0.05
**11**	>30	>200	-	4.52	4.79	4.52	4.52	0.27	0.00	0.00
**12**	8.0 ± 0.8	64.4 ± 4.2	8.1	5.10	5.25	4.99	5.27	0.15	0.11	0.18
**13**	10.0 ± 2.1	75.0 ± 13.8	7.5	5.00	5.46	4.91	5.01	0.46	0.09	0.01
**14**	>30	>200	-	4.52	4.57	4.50	4.46	0.05	0.02	0.06
**15**	9.5 ± 3.1	>200	>21.0	5.02	5.02	5.10	5.17	0.00	0.08	0.15
**16**	>30	>200	-	4.52	4.57	4.56	4.52	0.05	0.03	0.00
**17**	>30	>200	-	4.52	4.55	4.53	4.63	0.03	0.01	0.11
**18**	>30	>200	-	4.52	4.79	4.52	4.57	0.27	0.00	0.04
**19**	>30	>200	-	4.52	4.63	4.52	4.53	0.10	0.00	0.01
**20**	9.4 ± 2.2	>200	>21.3	5.03	4.90	5.01	5.07	0.13	0.01	0.04
**21**	>30	57.7 ± 1.1	-	4.52	4.92	4.55	4.28	0.40	0.03	0.24
**22**	>30	>200	-	4.52	4.91	4.55	4.78	0.04	0.33	0.10
**23**	>30	66.3 ± 6.0	-	4.52	4.36	4.51	4.46	0.83	0.01	0.07
**24**	>30	156.1 ± 15.0	-	4.52	5.03	4.53	4.47	0.51	0.00	0.05
**25**	>30	>200	-	4.52	5.00	5.12	5.14	0.12	0.01	0.01
**26**	>30	>200	-	4.52	4.58	4.12	4.10	0.06	0.40	0.43
**27**	12.2 ± 3.6	>200	>16.4	4.91	4.89	4.90	4.80	0.02	0.01	0.11
**28**	>30	>200	-	4.52	4.65	4.53	4.62	0.13	0.01	0.09
**29**	9.4 ± 1.8	>200	>21.3	5.03	4.47	4.51	4.60	0.05	0.52	0.42
**30**	>30	>200	-	4.52	4.82	4.52	4.47	0.30	0.00	0.06
**31**	13.3 ± 3.6	>200	>15.0	4.88	4.51	4.53	4.50	0.36	0.34	0.38
**32**	>30	>200	-	4.52	4.50	4.59	4.52	0.02	0.06	0.00
**33**	>30	>200	-	4.52	4.42	4.68	4.52	0.10	0.16	0.00
**34**	5.8 ± 0.7	>200	>34.5	5.24	5.38	5.24	5.23	0.15	0.00	0.00
**35**	10.9 ± 6.5	>200	>18.3	4.96	5.00	4.78	4.86	0.04	0.18	0.10
**36**	16.6 ± 1.0	42.0 ± 3.8	2.5	4.78	4.85	5.00	5.14	0.07	0.22	0.36
**37**	10.5 ± 8.3	14.2 ± 0.1	1.4	4.98	4.94	4.99	4.98	0.04	0.01	0.00
**38**	11.7 ± 7.0	>200	>17.1	4.93	4.85	4.92	4.88	0.08	0.01	0.05
**39**	5.5 ± 3.5	>200	>36.4	5.26	5.03	5.25	5.28	0.23	0.01	0.02
**40**	8.6 ± 2.1	>200	>23.3	5.07	5.07	5.08	5.12	0.00	0.02	0.06
**41**	13.4 ± 5.4	>200	>14.9	4.87	4.93	4.84	5.27	0.06	0.04	0.40
**42**	25.7 ± 12.2	>200	>7.8	4.59	4.48	4.54	4.52	0.11	0.05	0.07
**43**	7.7 ± 1.3	128.6 ± 5.2	16.7	5.11	4.99	5.01	5.15	0.12	0.10	0.03
**44**	11.6 ± 8.4	>200	>17.2	4.94	4.95	5.07	5.14	0.02	0.13	0.20
**45**	22.5 ± 18.8	123.4 ± 9.8	5.5	4.65	4.81	4.91	4.89	0.16	0.26	0.24
**46**	11.0 ± 2.3	>200	>18.2	4.94	4.93	4.96	4.87	0.03	0.00	0.09
**47**	>30	>200	-	4.52	4.46	4.50	4.55	0.06	0.02	0.03
**48**	>30	>200	-	4.52	4.60	4.55	4.53	0.08	0.02	0.00
**49**	>30	>200	-	4.52	4.47	4.56	4.52	0.05	0.04	0.00
**50**	>30	>200	-	4.52	4.60	4.69	4.61	0.08	0.17	0.09
**Bzd**	5.5 ± 1.4	190.2 ± 13.5	34.5	5.26	4.56	4.6	4.62	0.14	0.94	0.08

^1^: IC_50_ and CC_50_ for compounds **9**–**33** and **36**–**50** were reported at references [8,10,26]; ^2^: IC_50_, 50% inhibitory concentration against amastigote forms of *T. cruzi*; ^3^: CC_50_, 50% cytotoxic concentration in NCTC cells (ATCC, clone 929), NA, not active at 30 μM; ^4^: SI: selectivity index (ratio CC_50_/IC_50_); Bzd: benznidazole.

**Table 2:** Results obtained from the variations in the fragment distinctions (FD) using the default fragment size (4–7).

FD ^[1]^	FS ^[2]^	*q* ^2^ ^[3]^	SE ^[4]^	*r* ^2^ ^[5]^	SE_CV_^[6]^	HL ^[7]^	PCs ^[8]^
A/B	4–7	0.50	0.26	0.81	0.14	151	6
A/B/C		0.53	0.22	0.78	0.15	199	4
A/B/C/H		0.52	0.22	0.83	0.13	199	5
A/B/C/H/Ch		0.68	0.22	0.83	0.11	61	5
A/B/H/Ch/DA		0.54	0.24	0.85	0.13	199	6
A/B/H		0.48	0.21	0.79	0.15	151	4
A/B/C/Ch		0.55	0.26	0.77	0.15	83	4
A/B/DA		0.43	0.22	0.80	0.14	53	4
A/B/C/DA		0.54	0.24	0.84	0.13	257	5
A/B/H/DA		0.43	0.22	0.81	0.14	53	4
A/B/C/Ch/DA		0.60	0.22	0.88	0.11	61	6
A/B/C/H/DA		0.54	0.24	0.72	0.11	353	6
A/B/H/Ch/DA		0.75	0.22	0.88	0.16	61	6
A/B/H/Ch		0.49	0.26	0.81	0.14	151	6

^[1]^: FD, fragment distinction; ^[2]^: FS: fragment size; ^[3]^: *q*^2^, cross-validated coefficient; ^[4]^: SE, standard error; ^[5]^: *r*^2^, non-validated coefficient; ^[6]^: SE_CV_, standard error of cross-validation; ^[7]^: HL, hologram length; [^8]^: PCs, number of principal components.

**Table 3:** HQSAR results from different fragment sizes for the model generated previously (atoms, bonds, hydrogen bond donor, and acceptor).

FD ^[1]^	FS ^[2]^	*q* ^2^ ^[3]^	SE ^[4]^	*r* ^2^ ^[5]^	SE_CV_^[6]^	HL ^[7]^	PCs ^[8]^
A/B/H/Ch/DA	1–4	0.63	0.24	0.81	0.14	61	6
A/B/H/Ch/DA	2–5	0.54	0.24	0.82	0.14		5
A/B/H/Ch/DA	3–6	0.58	0.23	0.83	0.13		5
A/B/H/Ch/DA	4–7	0.75	0.22	0.88	0.16		6
A/B/H/Ch/DA	5–8	0.82	0.17	0.98	0.08		5
A/B/H/Ch/DA	6–9	0.45	0.25	0.88	0.12		6
A/B/H/Ch/DA	7–10	0.48	0.23	0.83	0.13		6

^[1]^: FD, fragment distinction; ^[2]^: FS, fragment size; ^[3]^: *q*^2^, cross-validated coefficient; ^[4]^: SE, standard error of validation; ^[5]^: *r*^2^, non-validated coefficient; ^[6]^: SE_CV_, cross-validated standard error; ^[7]^: HL, hologram length; ^[8]^: PCs, number of principal components.

**Table 4:** Statistical results for all CoMFA models obtained with and without the region focus.

	**No. Focus**	**d ^h^ = 0.3**				**w = 0.5**			
		**d ^i^ = 0.5**	**d = 1.0**	**d = 1.5**	**d = 2.0**	**d = 0.5**	**d = 1.0**	**d = 1.5**	**d = 2.0**
*q*^2^_LOO_ ^a^	0.64	0.76	0.83	0.81	0.73	0.82	**0.89**	0.86	0.62
SEP ^b^	0.18	0.15	0.13	0.14	0.14	0.13	**0.11**	0.12	0.18
*N* ^c^	5	5	6	5	5	6	**6**	6	3
*r* ^2 d^	0.99	0.99	0.99	0.99	0.99	0.99	**0.99**	0.99	0.93
SEE ^e^	0.02	0.01	0.01	0.03	0.03	0.02	**0.02**	0.02	0.08
S ^f^	0.53	0.49	0.50	0.52	0.52	0.53	**0.52**	0.55	0.58
E ^g^	0.77	0.51	0.50	0.48	0.48	0.48	**0.48**	0.45	0.42
		**w = 0.7**				**w = 0.9**			
		**d = 0.5**	**d = 1.0**	**d = 1.5**	**d = 2.0**	**d = 0.5**	**d = 1.0**	**d = 1.5**	**d = 2.0**
*q* ^2^_LOO_		0.72	0.81	0.28	0.05	0.12	0.68	0.08	0.28
SEP		0.16	0.13	0.24	0.24	0.28	0.18	0.27	0.23
*N*		4	6	2	2	5	6	1	1
*r* ^2^		0.99	0.99	0.89	0.45	0.90	0.93	0.83	0.44
SEE		0.03	0.04	0.10	0.23	0.10	0.08	0.13	0.21
S		0.53	0.54	0.70	0.53	0.55	0.67	0.41	0.00
E		0.47	0.46	0.30	0.48	0.45	0.33	0.59	1.00

^a^: *q*^2^_LOO_, leave-one-out cross-validated coefficient; ^b^: SEP, standard error of prediction; ^c^: *N*, number of PLS components; ^d^: *r*^2^, regression coefficient without validation; ^e^: SEE, standard non-cross validated error; ^f^: S, steric contribution; ^g^: E, electrostatic contribution. ^h^: w = weight; ^i^: d (Å) = distance between grid points; the best statistical model is highlighted in bold.

**Table 5:** Statistical results for all CoMSIA models obtained with and without region focus.

	**No. Focus**	**w ^h^ =0.3**				**w = 0.5**			
		**d ^i^ =0.5**	**d = 1.0**	**d = 1.5**	**d = 2.0**	**d = 0.5**	**d = 1.0**	**d = 1.5**	**d = 2.0**
*q* ^2^ *_LOO_* ^a^	0.45	0.54	0.61	0.62	0.43	0.69	**0.82**	0.61	0.61
SEP ^b^	0.23	0.21	0.19	0.19	0.23	0.21	**0.19**	0.19	0.29
*N* ^c^	4	5	4	5	4	6	**6**	5	4
*r* ^2 *d*^	0.92	0.89	0.91	0.91	0.88	0.93	**0.99**	0.89	0.60
SEE ^e^	0.05	0.10	0.09	0.09	0.10	0.10	**0.01**	0.10	0.20
S ^f^	0.33	0.39	0.41	0.43	0.37	0.45	**0.49**	0.46	0.47
H ^g^	0.67	0.61	0.59	0.57	0.63	0.57	**0.61**	0.53	0.53
		**w = 0.7**				**w = 0.9**			
		**d = 0.5**	**d = 1.0**	**d = 1.5**	**d = 2.0**	**d = 0.5**	**d = 1.0**	**d = 1.5**	**d = 2.0**
*q* ^2^ *_LOO_*		0.45	0.71	0.65	0.20	0.54	0.61	0.13	0.17
SEP		0.24	0.20	0.27	0.18	0.21	0.19	0.27	0.29
*N*		6	6	5	3	5	4	2	4
*r* ^2^		0.84	0.86	0.87	0.75	0.84	0.84	0.62	0.47
SEE		0.13	0.12	0.16	0.15	0.13	0.13	0.20	0.24
S		0.36	0.29	0.31	0.29	0.26	0.26	0.52	0.25
H		0.64	0.71	0.69	0.71	0.74	0.74	0.48	0.75

^a^: *q*^2^_LOO_, leave-one-out cross-validated coefficient; ^b^: SEP, standard error of prediction; ^c^: *N*, number of PLS components; ^d^: *r*^2^, regression coefficient without validation; ^e^: SEE, standard non-cross validated error; Field contribution, ^f^: H = hydrophobic; ^g^: S, steric contribution. ^h^: w = weight; ^i^: d (Å) = distance between grid points; the best model generated is highlighted in bold.

**Table 6:** Statistical parameters of the best-constructed 2D (HQSAR) and 3D (CoMFA and CoMSIA) models.

	HQSAR		CoMFA	CoMSIA
*q*^2^_LOO_ ^a^	0.82		0.89	0.82
SEE ^b^	0.17		0.11	0.19
*r* ^2 c^	0.98		0.99	0.99
SEEcv ^d^	0.08		0.02	0.01
*N* ^e^	5		6	6
Fdist ^f^	A/B/H/Ch/DA	S % ^i^	0.52	-
HL ^g^	61	E % ^j^	0.48	-
Fsize ^h^	5–8	S% ^i^	-	0.49
		H% ^k^	-	0.61
		Weight	0.9	0.8
		Distance	1.2	1.5
		*dq*^2^/*dr*^2*yy*′ m^	1.10	0.80

^a^: *q*^2^_LOO_, leave-one-out cross-validated coefficient; ^b^: SEE, standard error of calibration; ^c^: *r*^2^, non-cross validated coefficient; ^d^: SEE_CV_, leave-one-out cross-validation; ^e^: *N*, number of PLS components; ^f^: F-dist, fragment distinction (A, atoms; B, bonds; H, hydrogens, Ch, Chirality; DA, H-bonds donor, and acceptor); ^g^: HL, hologram length; ^h^: Fsize, fragment size. Field contribution: ^i^: S (steric); ^j^: E (electrostatic); ^k^: H (hydrophobic); ^m^: $dq^{2}/dr^{2yy^{\prime }}$: sensitivity index from the scrambling test.

**Table 7:** External validation of the HQSAR, CoMFA, and CoMSIA models.

Model	*q* ^2 [a]^	*r*^2^*_pred_* ^[b]^	*r*^2^*_m_* ^[c]^	Q^2^_F2_^[d]^	Q^2^_F3_^[d]^
HQSAR (A/B/H/Ch/DA)	0.82	0.90	0.82	0.91	0.98
CoMFA (d = 1.0 Å, w = 0.5)	0.89	0.94	0.84	0.95	0.99
CoMSIA (d = 1.0 Å, w = 0.5)	0.82	0.93	0.79	0.94	0.99

^[a]^ $q^{2}$: LOO cross-validation correlation coefficient; ^[b]^*r*^2^*_pred_*: external predictive potential of the model; ^[c]^*r*^2^*_m_*: external predictive potential of the model; ^[d]^ Q^2^: external predictive potential of the model.

## Data Availability

The additional data is available at Supplementary Materials.

## Supplementary Materials

The following are available online. Table S1. Experimental and predicted pIC50 for the test compounds; Table S2. Descriptors calculated for the compounds used in this study; Figure S1. Plot of Leverage versus Studentized residuals for (A) HQSAR, (B) CoMFA and (C) CoMSIA; Figure S2. Results from the cross-validation (LNO) of the obtained models: (A) HQSAR, (B) CoMFA and (C) CoMSIA; Figure S3. HRESIMS spectrum (positive mode) of compound **7**; Figure S4. ¹H NMR spectrum of compound **7** (δ, 300 MHz, CDCl_3_); Figure S5. ¹³C NMR spectrum of compound **7** (δ, 75 MHz, CDCl_3_) and Figure S6. IR spectrum of compound **7**.

## Sample Availability

Samples of the compounds are available from the authors.
