# Supplementary material for: Discovery of New Hits as Antitrypanosomal Agents by In Silico and In Vitro Assays Using Neolignan-Inspired Natural Products from Nectandra leucantha
Source: Molecules. 2021 Jul 6;26(14):4116. doi: 10.3390/molecules26144116 (PMC8306904; doi:10.3390/molecules26144116)
Supplement: Supplementary file 1 [file molecules-26-04116-s001.zip › molecules-1269154-supplementary.pdf]

Supplementary Material

Table S1. Experimental and predicted pIC_{50} for the test compounds

**Test set**		**HQSAR**		**CoMFA**		**CoMSIA**	
Compound	Exp. pIC_{50}	Pred. pIC_{50}	Residual	Pred. pIC_{50}	Residual	Pred. pIC_{50}	Residual
7	5.02	5.02	0.00	5.10	0.076	5.17	-0.145
8	4.52	4.57	-0.05	4.56	0.034	4.52	0.003
19	4.91	4.89	0.02	4.90	-0.010	4.80	0.112
23	4.52	4.51	0.01	4.53	0.011	4.50	0.026
29	4.82	4.72	0.10	4.71	-0.111	4.78	0.040
32	4.56	4.65	-0.09	4.63	0.064	4.51	0.051
34	5.24	5.38	-0.15	5.24	0.001	5.23	0.005
37	4.98	4.94	0.04	4.99	0.010	4.98	-0.005
42	4.59	4.48	0.11	4.54	-0.051	4.52	0.068
43	5.11	4.99	0.12	5.01	-0.104	5.15	-0.033

Table S2. Descriptors calculated for the compounds used in this study

cpd	pIC_{50}	TopoPSA	MW	LogP	PFP1	PFP 2	PFP 9	PFP 10	PFP 11	PFP12	PFP 18	PFP 19	PFP 20	PFP 178	PFP 182
1	4.79	36.92	368.20	6.10	1	1	1	1	1	1	1	1	1	1	1
2	4.52	36.92	382.21	6.49	1	1	1	1	1	1	1	1	1	1	1
3	4.52	36.92	366.18	5.80	1	1	1	1	1	1	1	1	1	1	1
4	5.10	53.99	368.16	5.97	1	1	1	1	1	1	1	1	1	1	1
5	5.00	53.99	382.18	6.36	1	1	1	1	1	1	1	1	1	1	1
6	4.52	36.92	354.18	5.70	1	1	1	1	1	1	1	1	1	1	1
7	5.02	36.92	402.18	7.60	1	1	1	1	1	1	1	1	1	1	1
8	4.52	36.92	420.23	7.14	1	1	1	1	1	1	1	1	1	1	1
9	4.52	36.92	358.21	6.30	1	1	1	1	1	1	1	1	1	1	1
10	4.52	36.92	372.23	6.69	1	1	1	1	1	1	1	1	1	1	1
11	4.52	36.92	386.25	7.08	1	1	1	1	1	1	1	1	1	1	1
12	5.03	36.92	370.21	6.39	1	1	1	1	1	1	1	1	1	1	1
13	4.52	47.92	330.18	5.98	1	1	1	1	1	1	1	1	1	1	1
14	4.88	36.92	344.20	5.91	1	1	1	1	1	1	1	1	1	1	1
15	4.52	53.99	372.19	6.56	1	1	1	1	1	1	1	1	1	1	1

16	4.52	53.99	386.21	6.95	1	1	1	1	1	1	1	1	1	1	1
17	5.12	47.92	326.15	5.38	1	1	1	1	1	1	1	1	1	1	1
18	4.06	47.92	326.15	5.77	1	1	1	1	1	1	1	1	1	1	1
19	4.91	36.92	340.17	5.71	1	1	1	1	1	1	1	1	1	1	1
20	4.52	71.06	372.16	4.50	1	1	1	1	1	1	1	1	1	1	1
21	4.52	77.38	376.19	4.21	1	1	1	1	1	1	1	1	1	1	1
22	4.52	88.38	362.17	3.92	1	1	1	1	1	1	1	1	1	1	1
23	4.52	77.38	376.19	3.85	1	1	1	1	1	1	1	1	1	1	1
24	4.52	71.06	316.09	3.60	1	1	1	1	1	1	1	1	1	1	1
25	4.52	79.44	458.10	6.05	1	1	1	1	1	1	1	1	1	1	1
26	4.52	47.92	328.17	5.68	1	1	1	1	1	1	1	1	1	1	1
27	4.52	36.92	340.17	5.31	1	1	1	1	1	1	1	1	1	1	1
28	4.52	58.92	298.12	4.33	1	1	1	1	1	1	1	1	1	1	1
29	4.82	57.15	356.16	4.46	1	1	1	1	1	1	1	1	1	1	1
30	4.52	36.92	312.14	4.93	1	1	1	1	1	1	1	1	1	1	1
31	4.55	64.99	340.13	4.62	1	1	1	1	1	1	1	1	1	1	1
32	4.56	53.99	354.15	4.92	1	1	1	1	1	1	1	1	1	1	1
33	5.37	71.06	382.14	5.61	1	1	1	1	1	1	1	1	1	1	1
34	5.24	71.06	396.16	6.00	1	1	1	1	1	1	1	1	1	1	1

35	4.96	27.69	310.16	5.31	1	1	1	1	1	1	1	1	1	1	1
36	4.78	47.92	328.17	5.68	1	1	1	1	1	1	1	1	0	1	1
37	4.98	47.92	328.17	5.68	1	1	1	1	1	1	1	1	1	1	1
38	4.93	36.92	342.18	5.61	1	1	1	1	1	1	1	1	1	1	1
39	5.26	46.15	450.24	7.15	1	1	1	1	1	1	1	1	1	1	1
40	5.07	57.15	434.21	6.92	1	1	1	1	1	1	1	1	1	1	1
41	4.87	46.15	448.22	6.85	1	1	1	1	1	1	1	1	1	1	1
42	4.59	36.92	300.14	4.66	1	1	1	1	1	1	1	1	1	1	1
43	5.11	38.69	296.14	5.37	1	1	1	1	1	1	1	1	1	1	1
44	4.94	36.92	416.20	6.55	1	1	1	1	1	1	1	1	0	1	1
45	4.65	38.69	296.14	5.37	1	1	1	1	1	1	1	1	1	1	1
46	4.96	36.92	416.20	6.55	1	1	1	1	1	1	1	1	0	1	1
47	4.52	36.92	300.14	4.66	1	1	1	1	1	1	1	1	1	1	1
48	4.52	46.15	406.18	5.90	1	1	1	1	1	1	1	1	1	1	1
49	4.52	36.92	326.15	5.14	1	1	1	1	1	1	1	1	1	1	1
50	4.52	46.15	406.18	5.90	1	1	1	1	1	1	1	1	1	1	1

IC_{50}: Half-maximal inhibitory concentration; **pIC_{50}:** -log IC_{50}; **TopoPSA:** Topological polar surface area; **MW**: Molecular weight; **LogP:** Partition coefficient of a molecule between aqueous and lipophilic phases (in general, water and octanol, respectively); **PFP:** PubChem fingerprint.

A)

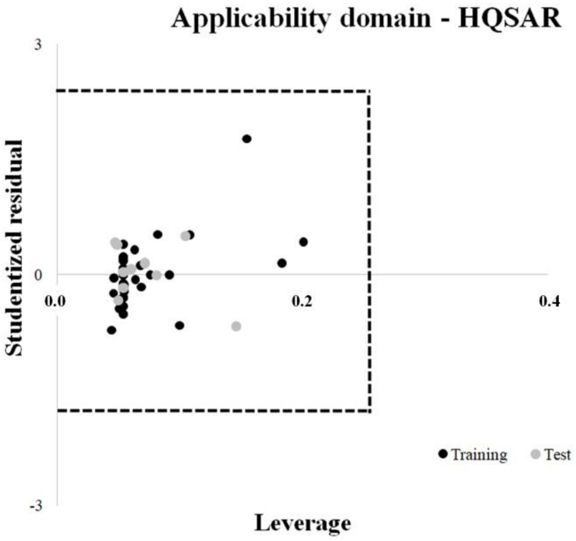

B)

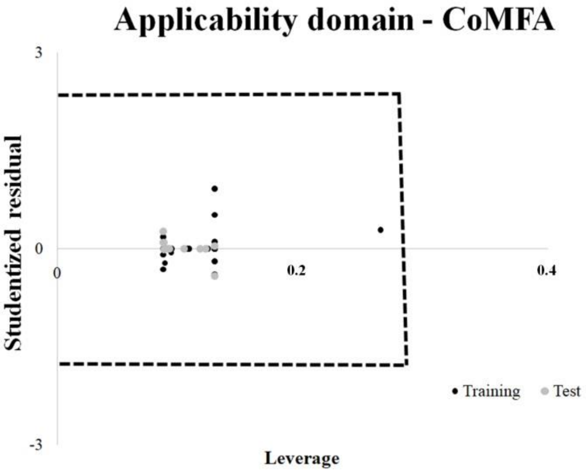

C)

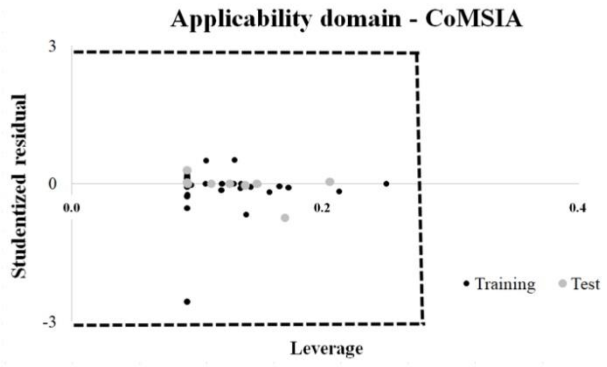

Figure S1. Plot of Leverage *versus* Studentized residuals for (A) HQSAR, (B) CoMFA and (C) CoMSIA (black dots represent the training set and grey dots represents the test compounds).

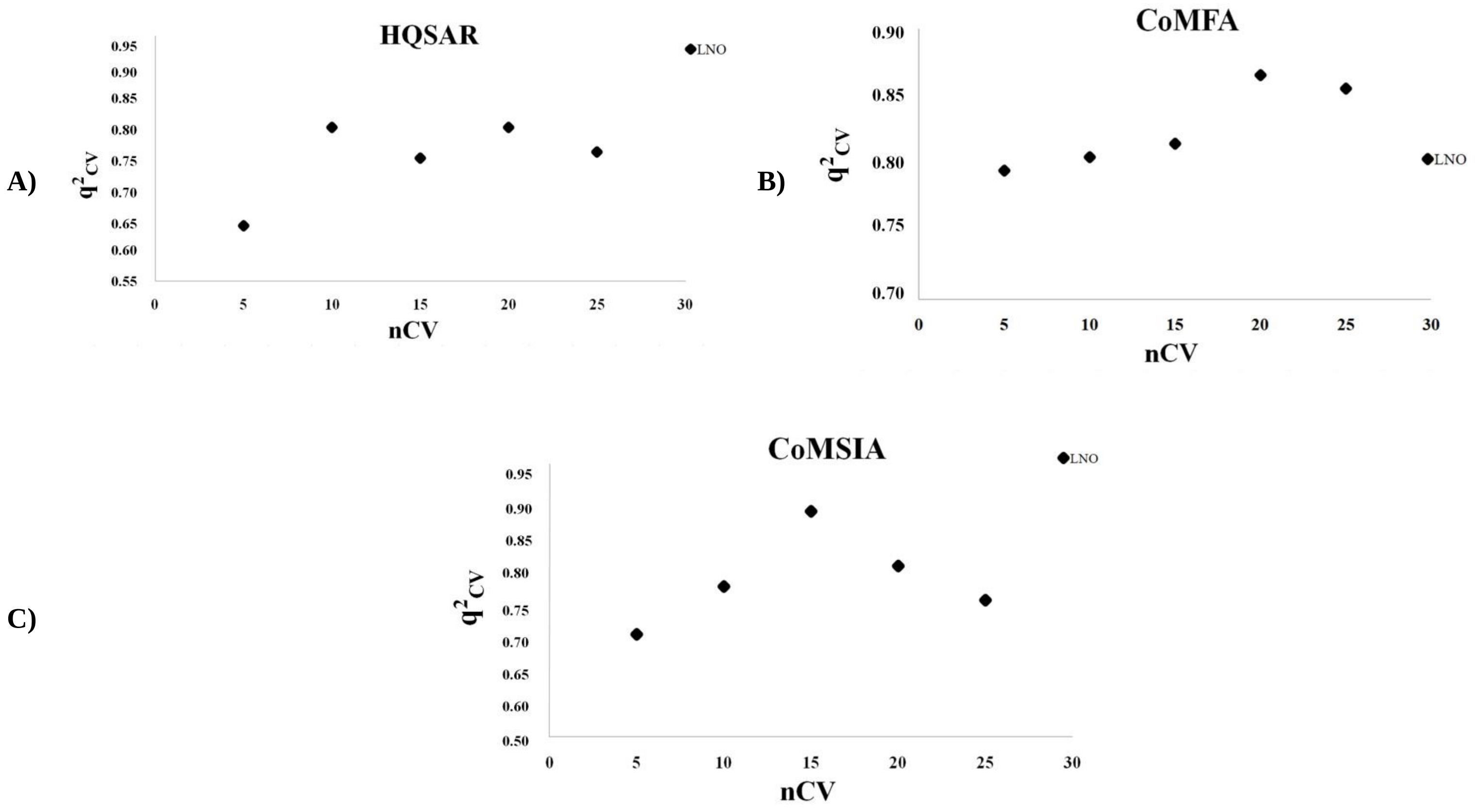

Figure S2. Results from the cross-validation (LNO) of the obtained models: (A) HQSAR, (B) CoMFA and (C) CoMSIA.

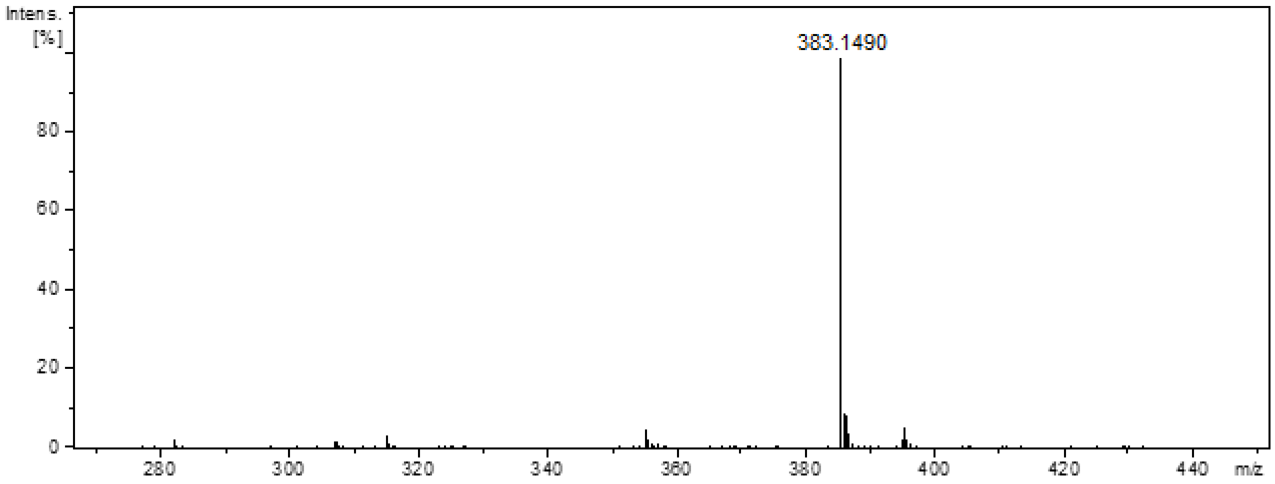

Figure S3. HRESIMS spectrum (positive mode) of compound **7.**

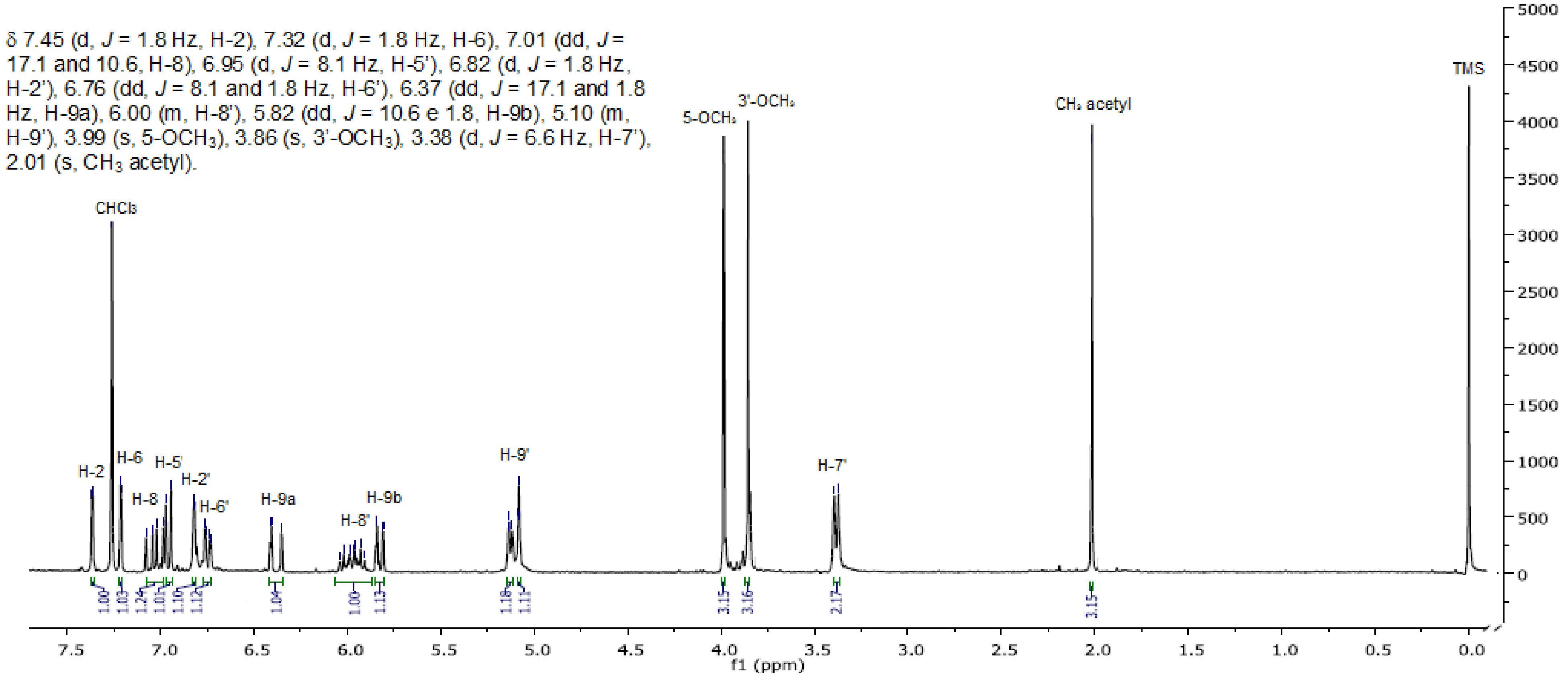

Figure S4. ¹H NMR spectrum of compound **7** (δ, 300 MHz, $CDCl_3$)**.**

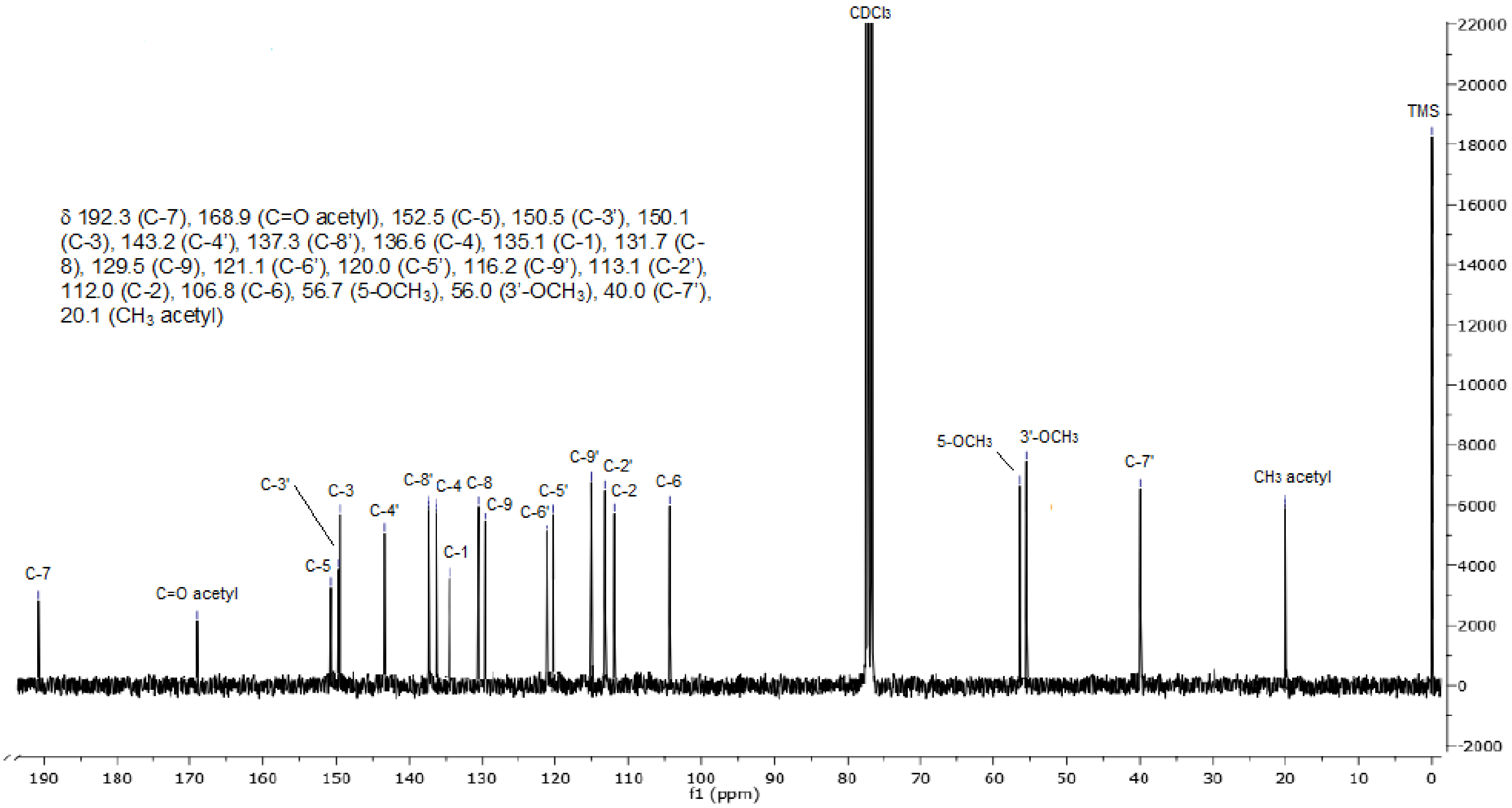

Figure S5. ^{13}C NMR spectrum of compound **7** (δ, 75 MHz, $CDCl_3$).

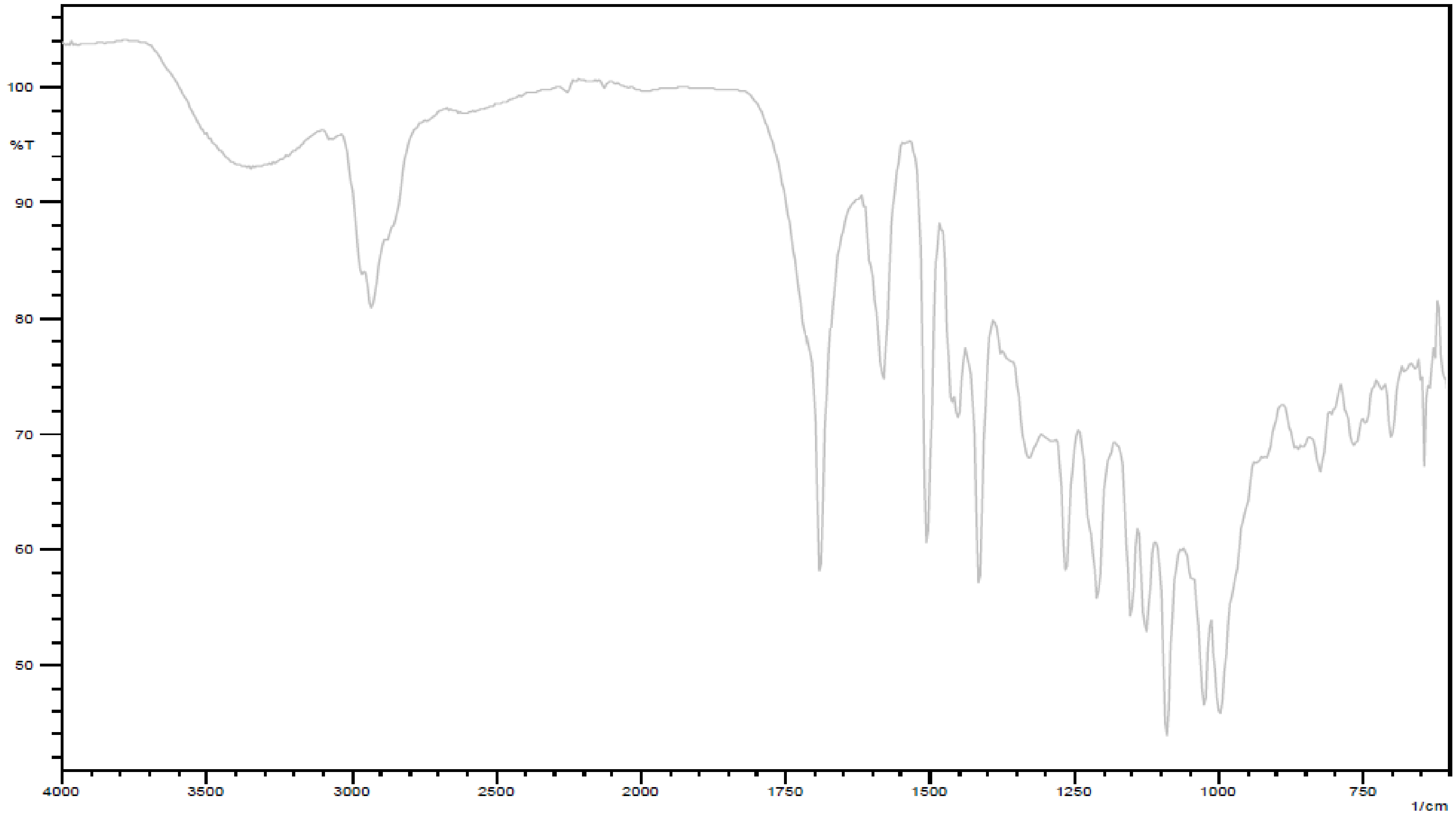

Figure S6. IR spectrum of compound **7.**
